# Comparing community *P. falciparum* infection prevalence measured via microscopy versus rapid diagnostic test

**DOI:** 10.1186/1475-2875-13-S1-P60

**Published:** 2014-09-22

**Authors:** Bonnie Mappin, Ursula Dalrymple, Ewan Cameron, Samir Bhatt, Daniel J Weiss, Peter W Gething

**Affiliations:** 1 University of Oxford, Oxford, UK

## 

Large scale mapping of *Plasmodium falciparum* infection prevalence, such as that undertaken by the Malaria Atlas Project, relies on opportunistic assemblies of data on infection prevalence arising from thousands of *P. falciparum* parasite rate (PfPR) surveys conducted worldwide. Variance in these data is driven by both signal - the true underlying pattern of infection prevalence - and a range of factors contributing to ‘noise’ - including sampling error, differing age ranges of subjects, and differing parasite detection methods. Whilst the former two have been addressed in previous maps, the effect of different diagnostic methods used to determine PfPR in different studies has not. In particular, the majority of PfPR data are based on positivity rates determined by either microscopy or rapid diagnostic test (RDT), and it is known that the sensitivity and specificity of these approaches are not equivalent. There is therefore a need for a method to quantitatively compare and adjust RDT- and microscopy-based prevalence estimates to a common standard prior to use in mapping. Here we estimate a relationship between RDT- and microscopy-derived PfPR using paired RDT and microscopy outcomes from sub-Saharan African populations. A total of 19 Demographic and Health Survey datasets from sub-Saharan Africa provide child diagnostic test results derived using both RDT and microscopy for each individual. We aggregated these prevalence estimates across administration zones (ADMIN1) and fitted a Bayesian probit regression to the microscopy- versus RDT-derived prevalence relationship. We employed an errors-in-variables approach to acknowledge sampling error in both the dependent and independent variable. In addition to the diagnostic outcome, several factors were extracted from the datasets in order to analyze their effect on observed malaria prevalence, sensitivity and specificity. These factors included: RDT type, fever status, recent ACT treatment, and estimated local population malaria prevalence.

We present results of stratified regression and analysis of variance analyses to establish the influence of these factors on measured prevalence, sensitivity and specificity. The fitted models can be applied to RDT-derived PfPR data to convert them to an estimate of the prevalence expected using microscopy, thereby standardizing the dataset and improving the signal-to-noise ratio. Additionally, our results provide insight into factors that influence the observed prevalence, sensitivity and specificity of different diagnostic techniques.

